# Harnessing hesperidin for brain metabolism: epigenetic signatures, cellular homeostasis, synaptic function, and neuroprotection in age-related diseases and neurodegenerative diseases

**DOI:** 10.3389/fnut.2025.1706146

**Published:** 2025-11-19

**Authors:** Wei Zhao, Wenwu Hong, Yingying Cao

**Affiliations:** 1 Department of Neurology, Taizhou Hospital of Integrated Traditional Chinese and Western Medicine, Wenling, China; 2 Department of Neurology, Tiantai People’s Hospital of Zhejiang Province (Tiantai Branch of Zhejiang Provincial People’s Hospital), Taizhou, China

**Keywords:** hesperidin, brain metabolism, synaptic function, neuroprotection, age-related diseases

## Abstract

Age-associated neurodegenerative disorders constitute a considerable global health concern, distinguished by a progressive deterioration in cognitive function, synaptic impairment, and disrupted cellular homeostasis. Recent research has identified hesperidin (HSP), a bioactive flavonoid predominantly present in citrus fruits, as a potentially valuable neurotherapeutic compound due to its diverse molecular mechanisms of action. This review aims to consolidate existing literature regarding HSP’s ability to influence epigenetic modifications, augment synaptic plasticity, and restore cellular equilibrium in the aging central nervous system. We investigate the manner in which HSP affects critical epigenetic markers, such as DNA methylation and histone modifications. Therefore it modulates gene expression essential for neuroprotection and lifespan extension. Furthermore, we examine the role of HSP in the preservation of synaptic integrity and neurotransmission, which are crucial for cognitive resilience in the face of age-related deterioration. The review further clarifies the antioxidant and anti-inflammatory properties of HSP, which together promote neuronal viability and alleviate neurodegenerative pathology. By synthesizing mechanistic insights and preclinical evidence, this article underscores the potential of HSP as a natural compound for both the prevention and adjunctive treatment of neurodegenerative disorders, thereby advocating for translational research aimed at realizing its full therapeutic efficacy.

## Introduction

The phenomenon of brain aging represents an unavoidable biological progression characterized by a gradual deterioration in cognitive capabilities including memory, attention, and executive function, concomitant with structural and biochemical alterations within the central nervous system (1). This progression is delineated by an accumulation of oxidative damage, mitochondrial dysfunction, disrupted proteostasis, neuroinflammation, synaptic degeneration, and modifications in neuronal signaling. Aging neural substrates are marked by an augmented generation of reactive oxygen species (ROS), which exceed the capacity of antioxidant defenses, thereby inducing oxidative stress, a principal contributor to neuronal impairment. Furthermore, mitochondrial bioenergetic dysfunction undermines adenosine triphosphate synthesis, which is vital for neuronal viability and operational integrity, and instigates apoptotic signaling cascades. Prolonged neuroinflammation, instigated by the activation of microglia and astrocytes, further intensifies neuronal damage through the secretion of pro-inflammatory cytokines and mediators (2, 3).

Hesperidin (HSP), a flavanone glycoside predominantly found in citrus fruits such as oranges, lemons, and grapefruits, encapsulates these neuroprotective attributes and has emerged as a compound of significant pharmacological relevance. From a chemical perspective, HSP is composed of hesperetin conjugated to the disaccharide rutinose, which imparts both hydrophilic and lipophilic characteristics that affect its bioactivity and metabolic pathways (4). The principal antioxidant and anti-inflammatory effects of HSP, together with its mitochondrial and proteostatic influences, are summarized later in the Mechanisms of Action section. Preclinical investigations have indicated that HSP and its metabolite HSD possess the capability to traverse the BBB, thereby accessing neuronal tissues where they manifest neuroprotective effects, including enhancement of cognitive functions, mitigation of amyloid-beta pathology, and reduction of oxidative and inflammatory injury (5).

In addition to these biochemical effects, HSP exerts an influence on gene expression via epigenetic mechanisms, including the modulation of DNA methylation, histone modifications, and microRNA regulation, which may play a significant role in its long-term neuroprotective properties (6). The objective of this investigation is to thoroughly examine the therapeutic potential of HSP in the modulation of cerebral metabolism, with a particular emphasis on its epigenetic signatures and their implications for cellular homeostasis, synaptic functionality, and neuroprotection. This narrative review aims to synthesize mechanistic and translational evidence regarding HSP’s neuroprotective potential. Rather than advocating for therapeutic application, our objective is to critically interpret the quality, consistency, and limitations of the current data. Where evidence is preliminary or inconsistent, this is explicitly discussed to maintain an analytical perspective.

### Methodological approach

This work was designed as a narrative review, aiming to synthesize and interpret current mechanistic and preclinical evidence regarding hesperidin’s neuroprotective potential rather than to conduct a systematic meta-analysis. A comprehensive literature search was performed across PubMed, Scopus, and Web of Science databases up to May 2025 using keywords such as “hesperidin,” “neuroprotection,” “epigenetics,” “oxidative stress,” “Alzheimer’s disease,” and “Parkinson’s disease.” Relevant experimental and clinical studies were selected based on scientific relevance, methodological rigor, and novelty. Because the objective was integrative rather than exhaustive, formal inclusion/exclusion criteria and bias assessment tools typical of systematic reviews were not applied, in accordance with the flexible framework appropriate for narrative reviews.

### Mechanisms of action

HSP exerts neuroprotective effects through a limited set of complementary mechanisms that together support cellular resilience. At the core are antioxidant actions (direct radical scavenging and upregulation of endogenous enzymes via Nrf2), and anti-inflammatory effects (attenuation of NF-κB signaling and cytokine release). HSP further preserves mitochondrial function by maintaining membrane potential, improving electron-transport efficiency, and supporting PGC-1α–mediated biogenesis. In parallel, HSP promotes autophagy and proteostasis (via modulation of PI3K/Akt/mTOR and related pathways), which facilitates clearance of dysfunctional organelles and aggregates. Finally, HSP influences epigenetic regulation (DNA methylation, histone modification and microRNA expression), providing a plausibly durable layer of transcriptional modulation. Together, these interconnected mechanisms provide a compact framework for interpreting the disease-specific findings described below (7–11).

### Chemical properties and pharmacokinetics of HSP

HSP is classified as a flavanone glycoside, specifically identified as 3′,5,7-trihydroxy-4′-methoxyflavanone-7-rhamnoglucoside in chemical nomenclature. The molecular architecture of this compound comprises the aglycone hesperetin, which is covalently bonded to the disaccharide rutinose, consisting of rhamnose and glucose, at the 7-position on the flavonoid scaffold. This glycosidic bond plays a pivotal role in determining its physicochemical characteristics, particularly its solubility and absorption kinetics. The existence of several hydroxyl groups enhances its antioxidant capabilities, facilitating the processes of free radical interception and metal ion chelation (12, 13). HSP is predominantly located in citrus fruits, with a notable concentration in the peels, albedo (the white inner layer of the peel), and pulp of oranges, lemons, grapefruits, as well as other citrus species. These natural sources represent the principal dietary pathways through which individuals ingest HSP. Notwithstanding its notable biological activity, HSP exhibits diminished bioavailability upon oral administration. This constraint is predominantly attributable to its limited solubility in aqueous environments and the presence of the glycosidic sugar moiety, which impedes passive diffusion through the intestinal epithelial barrier. Following ingestion, HSP arrives at the colon, where the gut microbiota enzymatically cleaves the glycosidic bond, thereby liberating the aglycone hesperetin. Hesperetin possesses enhanced lipophilicity, which facilitates its more efficient absorption into enterocytes. Within the intestinal epithelial cells and hepatic tissues, hesperetin undergoes comprehensive phase II metabolic processes characterized by conjugation reactions, including glucuronidation, sulfation, and methylation. These metabolic alterations augment its hydrophilicity, thereby promoting systemic circulation and subsequent renal or biliary excretion. The resultant metabolites, predominantly hesperetin glucuronides and sulfates, maintain biological activity and significantly contribute to the overarching pharmacological effects associated with HSP (14, 15). Noteworthy, several formulation strategies have been investigated to enhance systemic exposure and brain delivery. Nanoparticle-based systems (e.g., chitosan, PLGA, and gold nanoparticles) have been shown to improve solubility, stability, and controlled release of HSP, significantly increasing brain accumulation in animal models. Lipid-based carriers, such as liposomes and solid lipid nanoparticles, improve membrane permeability and protect HSP from enzymatic degradation. Additionally, phospholipid complexes (phytosomes) and co-administration with bioactive enhancers like piperine, quercetin, or vitamin C have demonstrated improved pharmacokinetic profiles and antioxidant efficacy. These approaches collectively suggest practical translational routes to optimize HSP delivery and therapeutic potential for neurodegenerative disorders (16–18).

With respect to the permeability of the BBB, HSP inherently possesses a constrained ability to traverse the BBB owing to its hydrophilic glycoside configuration, which impedes passive diffusion into cerebral tissues. Conversely, its aglycone variant, hesperetin, demonstrates enhanced lipophilicity, thereby facilitating partial ingress through the BBB. This particular attribute permits hesperetin to manifest neuroprotective properties by modulating oxidative stress, inflammation, and neuronal signaling within the central nervous system (19, 20). Empirical investigations have substantiated hesperetin’s capacity to access brain tissues, wherein it may exert influence over pathways pertinent to neurodegenerative disorders, cognitive processes, and neuroinflammatory responses (21, 22). The extent of BBB permeability is modulated by the interactions of hesperetin with efflux transporters and its enzymatic metabolism within the brain, which remain focal points of contemporary research. Despite evidence that its aglycone metabolite, hesperetin, can cross the BBB, the parent compound hesperidin shows limited permeability and poor systemic bioavailability following oral administration (23, 24). Rapid intestinal metabolism and first-pass conjugation significantly reduce circulating levels of the active form. Consequently, the concentrations achieved in the brain after conventional dosing are likely much lower than those used in most *in vitro* or animal experiments. These pharmacokinetic and BBB constraints represent a key translational obstacle, underscoring the gap between preclinical efficacy and achievable human exposure.

In conclusion, HSP represents a flavanone glycoside that is prevalent in citrus fruits, distinguished by a glycosidic bond that influences its solubility and absorption characteristics. Its bioavailability is constrained; however, it can be enhanced through microbial metabolism into hesperetin, which subsequently undergoes conjugative metabolic processes within the organism. Although HSP itself exhibits limited ability to traverse the BBB, hesperetin possesses the capacity to penetrate this barrier, thereby facilitating neuroprotective effects, which underscores the therapeutic implications of HSP and its metabolic derivatives.

### Epigenetic mechanisms modulated by HSP

HSP has been demonstrated to modulate a variety of epigenetic mechanisms, thereby influencing gene expression without modifying the intrinsic DNA sequence. One of the principal epigenetic modifications influenced by HSP is DNA methylation. Research indicates that HSP may affect the activity of DNA methyltransferases, which are enzymes responsible for the addition of methyl groups to cytosine residues within DNA, resulting in alterations in gene expression patterns, particularly in genes associated with inflammation, oxidative stress, and the regulation of the cell cycle (6, 25).

Through the modulation of DNA methylation, HSP could potentially aid in the restoration of normal expression levels of tumor suppressor genes or the downregulation of oncogenes, thereby contributing to its chemopreventive attributes. Furthermore, HSP exerts effects on histone modifications, which are critical for regulating chromatin architecture and gene accessibility. It has been documented that HSP influences histone acetylation and methylation by modulating the activity of histone acetyltransferases, histone deacetylases, and histone methyltransferases (26).

Through the inhibition of histone deacetylases, HSP is capable of fostering a chromatin structure that is more relaxed, thereby facilitating the transcription of genes that counteract oxidative stress and inflammation. This epigenetic modification is essential for the preservation of cellular homeostasis and the prevention of disease development. Moreover, HSP may influence the expression of non-coding RNAs, such as microRNAs (miRNAs), which are pivotal post-transcriptional regulators of gene expression. Specific miRNAs that are modulated by HSP have been associated with the regulation of pathways pertinent to apoptosis, autophagy, and metabolic processes (27, 28).

By modulating miRNA expression profiles, HSP plays a crucial role in optimizing cellular responses to stress while sustaining physiological equilibrium. Therefore, through the modulation of DNA methylation, histone modifications, and the expression of non-coding RNAs, HSP demonstrates substantial epigenetic effects that underpin its protective functions against oxidative stress, inflammation, and metabolic disturbances. For example, studies in RAW 264.7 cells demonstrated that hesperetin suppresses acetylation of NF-κB p65 (RelA) by inducing SIRT1 expression, which is a known histone deacetylase (29). Also, hesperidin has been shown to upregulate miR-132 in certain cancer models, modulating expression of target genes through epigenetic-like regulation (30).

These epigenetic changes do more than shift gene expression in isolation; they interact (“cross-talk”) with other cellular systems, such as autophagy, mitochondrial function, and inflammation. For instance, upregulation of SIRT1 can lead to enhanced autophagy and mitochondrial biogenesis via activation of AMPK and PGC-1α pathways, improving energy metabolism and reducing oxidative stress (31). Similarly, suppression of NF-κB acetylation via histone deacetylation may reduce pro-inflammatory cytokines, which in turn can lessen mitochondrial damage and promote cellular survival (32).

In addition, microRNAs regulated by hesperidin (or similar compounds) appear to target components of inflammatory and mitochondrial pathways. For example, miR-132 regulates neurite growth and synaptic plasticity, while miR-155 is often implicated in neuroinflammation (33–35). Even though direct in-vivo data linking hesperidin’s modulation of miR-132 or miR-155 (in brain tissue) with mitochondrial/autophagy effects is sparse, the pattern in related studies suggests this is a promising direction.

Together, these findings support the idea that hesperidin’s novelty as a multi-target modulator rests partly on its ability to reshape epigenetic regulation, which then influences autophagy, mitochondrial function, and inflammatory response in a coordinated way, not as separate, isolated mechanisms.

### Role of HSP in maintaining cellular homeostasis

As summarized in the Mechanisms of Action section, HSP enhances endogenous antioxidant defenses and mitigates oxidative and inflammatory stress. Building upon these general effects, the following section details how these mechanisms contribute to maintaining mitochondrial and proteostatic homeostasis.

Furthermore, HSP influences signaling cascades such as nuclear factor erythroid 2–related factor 2 (Nrf2), recognized as a principal regulator of antioxidant gene expression, thus further bolstering cellular defense systems against oxidative threats (36, 37). Beyond the implications of oxidative stress, HSP is instrumental in sustaining mitochondrial functionality and bioenergetics. Mitochondria serve as both sources and targets for ROS, and their dysfunction is associated with a myriad of pathological conditions, including neurodegenerative diseases, metabolic disorders, and the aging process. HSP conserves mitochondrial membrane potential, diminishes mitochondrial ROS production, and inhibits the opening of the mitochondrial permeability transition pore (MPTP), thus safeguarding mitochondrial integrity (38). Furthermore, it enhances ATP synthesis by augmenting the efficiency of the electron transport chain and fostering mitochondrial biogenesis through the activation of signaling pathways involving PGC-1α (peroxisome proliferator-activated receptor gamma coactivator 1-alpha), a pivotal regulator of mitochondrial replication and functionality (39).

By preserving mitochondrial homeostasis, HSP guarantees an adequate supply of cellular energy, which is crucial for the normal functioning and survival of cells under conditions of stress.

Moreover, HSP exerts a significant influence on autophagy and proteostasis, both of which are essential for the maintenance of cellular quality control and homeostasis. Autophagy constitutes the mechanism through which cells dismantle and recycle compromised organelles and misfolded proteins, thereby averting their toxic accumulation within the cellular milieu. Research indicates that HSP can stimulate autophagy by modulating critical signaling cascades such as PI3K/Akt/mTOR, which are pivotal in the initiation and advancement of autophagic flux (7, 40). This facilitation of autophagy aids in the elimination of dysfunctional mitochondria (mitophagy) and protein aggregates, thereby upholding proteome integrity and mitigating cellular stress. In addition, the anti-inflammatory properties of HSP complement its role in proteostasis by curtailing chronic inflammatory responses that may intensify proteotoxic stress. Collectively, these mechanisms contribute to the preservation of cellular homeostasis and promote cellular survival in the presence of diverse stressors. In conclusion, HSP plays a pivotal role in cellular homeostasis by augmenting antioxidant mechanisms to mitigate oxidative stress, safeguarding and enhancing mitochondrial functionality and bioenergetics, as well as facilitating autophagy and proteostasis to sustain the integrity of proteins and organelles. Collectively, these synergistic effects render HSP a promising natural compound for the protection of cells against stress-induced damage and the preservation of physiological equilibrium.

### Effects of HSP on synaptic function and plasticity

HSP has exhibited auspicious effects on synaptic functionality and plasticity, which are essential for the processes of learning, memory formation, and overall cognitive well-being. A principal mechanism through which HSP manifests its neuroprotective qualities is by modulating neurotransmitter systems. Empirical studies have revealed that HSP can affect critical neurotransmitters, including gamma-aminobutyric acid (GABA), glutamate, dopamine, and acetylcholine, all of which are integral to both excitatory and inhibitory synaptic transmission. For instance, research has indicated that HSP elevates levels of acetylcholine and amplifies cholinergic signaling, consequently enhancing cognitive functions that are often compromised in neurodegenerative disorders such as AD (41, 42). Beyond the modulation of neurotransmitters, HSP plays a significant role in the augmentation of synaptic plasticity, which is fundamental to the brain’s ability to adapt and reorganize in response to various experiences. Experimental models of aging and neurodegeneration that have been administered HSP demonstrate enhancements in long-term potentiation (LTP), which serves as a cellular correlate for memory and learning (43). Empirical studies further corroborate these observations, revealing that the administration of HSP results in enhanced performance in tasks pertaining to memory and spatial learning, thereby suggesting its potential efficacy in reversing cognitive deficits (42). The neuroprotective properties attributed to HSP are additionally associated with its modulation of neurotrophic factors and the corresponding signaling cascades. Evidence suggests that HSP facilitates the upregulation of brain-derived neurotrophic factor (BDNF), which serves as a crucial regulator of synaptic plasticity and neuronal viability, via the activation of specific signaling pathways (44, 45). Through the enhancement of BDNF signaling, HSP not only fosters synaptic development and restructuring but also bolsters cellular resilience in the face of age-associated and pathological challenges. This intricate modulation of synaptic integrity by HSP underscores its potential as a therapeutic agent in the preservation of cognitive function and the mitigation of neurodegenerative disorders.

### Preclinical and clinical evidence supporting HSP’S neuroprotective effects in aging

Preclinical investigations utilizing aged animal models have elucidated that HSP markedly enhances cognitive function, diminishes oxidative stress, and alleviates neuroinflammation—critical elements implicated in age-associated neurodegeneration. These observed effects are attributable to its capacity to augment antioxidant enzyme activity, modulate signaling cascades, and sustain neuronal integrity. Although the clinical evidence is somewhat limited, it lends support to HSP’s potential efficacy in ameliorating memory, mood, and overall cerebral function in geriatric populations. Collectively, this burgeoning corpus of preclinical and clinical evidence underscores HSP as a promising phytochemical for the prevention or attenuation of cognitive decline and neurodegenerative disorders linked to the aging process. Additional rigorously designed clinical trials are imperative to substantiate and broaden these findings for therapeutic application. The investigation examined the ramifications of Altermor®, a nutritional supplement comprising HSP, diosmin, and proanthocyanidins, on cognitive function, equilibrium, fatigue, and overall quality of life among geriatric individuals. In a randomized, crossover-controlled pilot study involving 36 subjects, participants administered Altermor® exhibited notable enhancements in cognitive function (particularly in the domain of attention) and a diminished risk of falls when juxtaposed with a control group. No statistically significant difference was observed between the two dosage levels evaluated (1 stick/day versus 2 sticks/day). The results imply that Altermor® may contribute to the augmentation of cognitive abilities and the mitigation of fall risk in the elderly population, thereby necessitating further inquiry into cognitive deficits (46). The investigation examined HSP as a prospective therapeutic intervention for sarcopenia, which is defined as the age-associated decline in muscle mass and strength that detrimentally impacts the quality of life in elderly populations. Employing aged murine models that correspond to human subjects over the age of 70, a regimen of daily HSP administration over a duration of eight weeks resulted in significant enhancements in muscle mass, strength, and muscle fiber hypertrophy. Furthermore, HSP contributed to the reestablishment of immune equilibrium that is often disrupted by the aging process, while concurrently ameliorating sarcopenia through the activation of specific cellular signaling cascades associated with muscle hypertrophy. These findings indicate that HSP may serve as a promising therapeutic agent for sarcopenia by sustaining immune homeostasis (47).

Another investigation assessed the impact of a citrus and pomegranate complex (CPC) on physical fitness, psychological well-being, and hematological biomarkers in a cohort of healthy elderly individuals aged between 60 and 75 years. Following a four-week regimen of CPC supplementation, participants exhibited notable enhancements in handgrip strength as well as cognitive functions including reasoning, memory retention, and attentional capacity. Furthermore, a reduction in oxidative stress levels was observed. Nevertheless, no statistically significant alterations were detected in overall senior fitness, the majority of quality of life metrics, or additional hematological markers. The findings imply that CPC may serve to augment physical strength and cognitive function among older adults, thereby necessitating further scholarly exploration (48). An investigation examined the application of HSP, a naturally occurring phytochemical, as a sustainable stabilizer for ethylene-norbornene copolymers within the domain of polymer technology. Empirical evaluations demonstrated that HSP significantly enhanced surface energy, mechanical characteristics, and resistance to aging of the polymeric material. Furthermore, it contributed positively to the attributes of Topas-silica composites and functioned as a dye or chromatic indicator, thereby indicating prospective applications in sectors such as food packaging. Given the scarcity of scholarly research concerning the utilization of HSP in this context, the results underscore its viability as a natural substitute for synthetic stabilizers in polymer formulations (49).

Another investigation examined the potential of HSP to mitigate age-associated aortic stiffness and the stiffening impacts attributable to perivascular adipose tissue (PVAT) and advanced glycation end-products (AGEs). In geriatric mice, arterial stiffness and the accumulation of AGEs were markedly elevated compared to their younger counterparts. A four-week regimen of HSP administration ameliorated these conditions, reducing arterial stiffness and AGE concentrations to levels comparable to those observed in young mice. Furthermore, HSP diminished the stiffening of arteries induced by PVAT. These findings imply that HSP may serve as an effective intervention against age-related vascular stiffening by addressing the accumulation of PVAT and AGEs (50). An investigation elucidated the impact of citrus flavanones naringenin and HSP on hepatic antioxidant functionality and membrane composition in rats aged 24 months. Throughout a duration of four weeks, both compounds enhanced the activity of antioxidant enzymes and diminished markers of oxidative stress in the liver when compared to untreated aged control subjects. Naringenin demonstrated superior efficacy relative to HSP in augmenting various antioxidant enzymes and elevating liver glutathione concentrations. Both flavanones positively influenced membrane phospholipid profiles and mitigated markers of hepatic damage within the bloodstream, all while preserving the structural integrity of liver tissue. Collectively, the results indicate that NAR and HSP exert protective, anti-aging properties on hepatic function (51).

Another research examined the neuroprotective properties of HSP utilizing an aged murine model of PD that was induced through the administration of 6-hydroxydopamine (6-OHDA). The administration of HSP (50 mg/kg over a duration of 28 days) resulted in enhancements in cognitive function and a diminution of depressive-like behaviors. Furthermore, it reinstated the activities of antioxidant enzymes, augmented the levels of dopamine and its metabolites within the cerebral tissue, and mitigated oxidative stress instigated by 6-OHDA. These findings indicate that HSP may confer neuroprotection and has the potential to serve as a therapeutic intervention for PD (52). An investigation evaluated commercial flavonoids for their potential anti-aging properties utilizing a yeast model and identified that HSP, derived from citrus, significantly prolonged yeast lifespan at minimal concentrations. HSP diminished the levels of ROS and downregulated the expression of the UTH1 gene, with the SKN7 gene implicated in the mechanism of lifespan extension. Furthermore, it enhanced the activity of the anti-aging gene Sir2 (SIRT1) alongside various antioxidant genes. These findings imply that the anti-aging properties of HSP in yeast are associated with the inhibition of ROS and the modulation of specific genetic pathways, whereas its structurally related compound, hesperetin, exhibited no comparable effects (53).

The investigation examined the role of hesperetin (HST) as an activator of the longevity-associated gene Cisd2. In senescent mice, the administration of dietary HST resulted in an upregulation of Cisd2 expression, an extension of healthspan, and an amelioration of age-related metabolic and organ deterioration. The observed anti-aging effects appeared to be contingent upon Cisd2, with the treated mice demonstrating a gene expression profile indicative of a more youthful state. These findings imply that HST may represent a viable strategy for decelerating the aging process and enhancing longevity through the activation of Cisd2 (54). A research examined the neuroprotective properties of HSP supplementation in conjunction with alternate day fasting (ADF) in middle-aged rats. The group of rats subjected to ADF with HSP demonstrated enhanced antioxidant defenses, diminished oxidative stress and inflammation, improved mitochondrial functionality, and superior brain tissue integrity relative to the control group. Furthermore, this combination resulted in an increase in autophagy gene expression, which contributes to the protection of neurons from aging-associated damage. These results indicate that the combination of HSP and ADF might synergistically mitigate oxidative stress and neurodegeneration, thereby potentially decreasing the risks of metabolic and cognitive decline associated with aging (55).

While current preclinical findings are consistent and generally supportive, several limitations should be recognized. Animal models vary considerably in strain, age, dose, route, and duration of HSP administration, which complicates comparison and generalization of results. Moreover, most published studies report positive outcomes, suggesting possible publication bias. Human data remain preliminary, such as the Altermor® pilot trial (36 participants), and provide only early indications of clinical relevance without sufficient statistical power to infer efficacy (46). These factors collectively underscore the need for larger, standardized, and independently replicated studies before firm conclusions can be drawn about translational value.

Overall, HSP demonstrates significant neuroprotective characteristics, substantiated by both preclinical investigations and emerging clinical findings, rendering it an intriguing candidate for addressing age-associated cognitive decline and neurodegenerative conditions. Its properties as an antioxidant, along with its anti-inflammatory and neuro-regulatory effects, play a crucial role in preserving cerebral health throughout the aging process. Ongoing research, particularly comprehensive clinical trials, is imperative to thoroughly ascertain the efficacy of HSP and to enhance its therapeutic potential within aging demographics. It should be noted that most available studies are preclinical, and differences in experimental design or publication bias may overestimate the apparent consistency of results (Table 1).

**Table 1:** The beneficial effects of hesperidin on brain health in age-related diseases and neurodegenerative diseases.

Dosage of hesperidin	Duration of study	Model (*In vitro* or *in vivo*)	Main effects	Mechanism of effect	Ref
1 stick/day and 2 sticks/day of Altemor® (contains hesperidin, diosmin, proanthocyanidins)	8 weeks per dose with 4-week washout (Total: 20 weeks)	*In vivo* (human clinical trial – elderly participants)	- Improved cognitive function (attention, learning, memory) - Reduced risk of falling	Likely due to improved blood microcirculation and neuroprotective effects via antioxidant and anti-inflammatory activity of flavonoids (hesperidin and others)	(46)
Daily administration (exact dose not specified)	8 weeks	*In vivo* (aged mice, 22–26 months old)	- Increased muscle mass and strength - Increased muscle fiber size - Restored immune homeostasis (corrected M1/M2 macrophage ratio)	Regulation of AKT/mTOR/FoxO3a signaling pathway via increased IGF-1 expression; modulation of inflammaging and immune balance	(47)
Part of citrus and pomegranate complex (exact hesperidin dose not specified)	4 weeks	*In vivo* (healthy elderly humans, 60–75 years)	- Improved handgrip strength - Improved thinking, memory, learning, concentration - Reduced plasma malondialdehyde (oxidative stress marker)	Antioxidant effects reducing oxidative stress, possibly improving endothelial function and cognitive performance	(48)
Not specified (used as additive in polymer composites)	Not specified (laboratory material testing timeline)	*In vitro* (polymer composite system)	- Improved surface and mechanical properties - Increased aging resistance - Acted as dye/indicator	Acts as an antioxidant stabilizer: prevents degradation by scavenging free radicals during aging/processing	(49)
Not specified (administered to old mice)	4 weeks	*In vivo* (C57BL/6 mice; young and old)	- Reduced aortic pulse wave velocity (aPWV) - Reduced intrinsic mechanical stiffness of aorta - Reversed PVAT AGE accumulation	Inhibition/reversal of advanced glycation end-products (AGE) accumulation in perivascular adipose tissue (PVAT), reducing arterial stiffness associated with aging	(50)
15 mg/kg orally, once daily	4 weeks	*In vivo* (24-month-old male Wistar rats)	- Increased antioxidant enzyme activity (CAT, GR) - Reduced lipid peroxidation (TBARS) - Improved membrane phospholipid composition (favoring n-3 PUFA) - Reduced liver enzyme serum levels (ALT, AST)	Enhancement of liver antioxidant defense system, reduction of oxidative stress, and improvement of membrane lipid quality	(51)
50 mg/kg (oral)	28 days (4 weeks)	*In vivo* (aged mice with 6-OHDA-induced Parkinson’s model)	- Prevented memory impairment and depressive-like behavior - Restored dopamine and its metabolites in the striatum - Reduced oxidative stress (ROS) - Improved antioxidant enzyme activities (GPx, CAT)	Antioxidant action: reduced ROS, restored glutathione system, and increased antioxidant enzyme activity; protected dopaminergic neurons	(52)
5 μM and 10 μM	Not explicitly stated (typical yeast replicative lifespan assay: ~days to weeks)	*In vitro* (K6001 yeast strain)	- Extended replicative lifespan in yeast - Reduced ROS levels - Increased SIRT1 and SOD expression	Inhibition of ROS Downregulation of UTH1 gene Activation of SIRT1 pathway SKN7 gene involvement	(53)
Not precisely specified (dietary supplementation in aged mice)	Long-term (late-life administration; likely weeks to months)	*In vivo* (naturally aged mice; tissue-specific Cisd2 knockout mice)	- Prolonged healthspan - Ameliorated metabolic decline and organ senescence - Restored youthful gene expression patterns	Activation of Cisd2 gene expression (longevity-associated) Cisd2-dependent regulation of metabolism and aging-related pathways	(54)
Not specified (regular administration during study)	Not explicitly stated (typical ADF studies last several weeks)	*In vivo* (middle-aged male Wistar rats, 12–15 months)	- Enhanced antioxidant defense (increased FRAP, GSH, SOD, catalase) - Reduced oxidative stress markers (MDA, PCO, AOPP, NO) - Lowered inflammation (IL-6, TNF-α) - Increased autophagy (Beclin gene expression) - Improved mitochondrial electron transport chain efficiency - Neuroprotection and reduced neurodegeneration	Synergistic antioxidant and anti-inflammatory effects during ADF Enhancement of autophagy and mitochondrial function to reduce oxidative stress and neurodegeneration	(55)

### Preclinical and clinical evidence supporting HSP’S neuroprotective effects in AD

**Figure 1:**
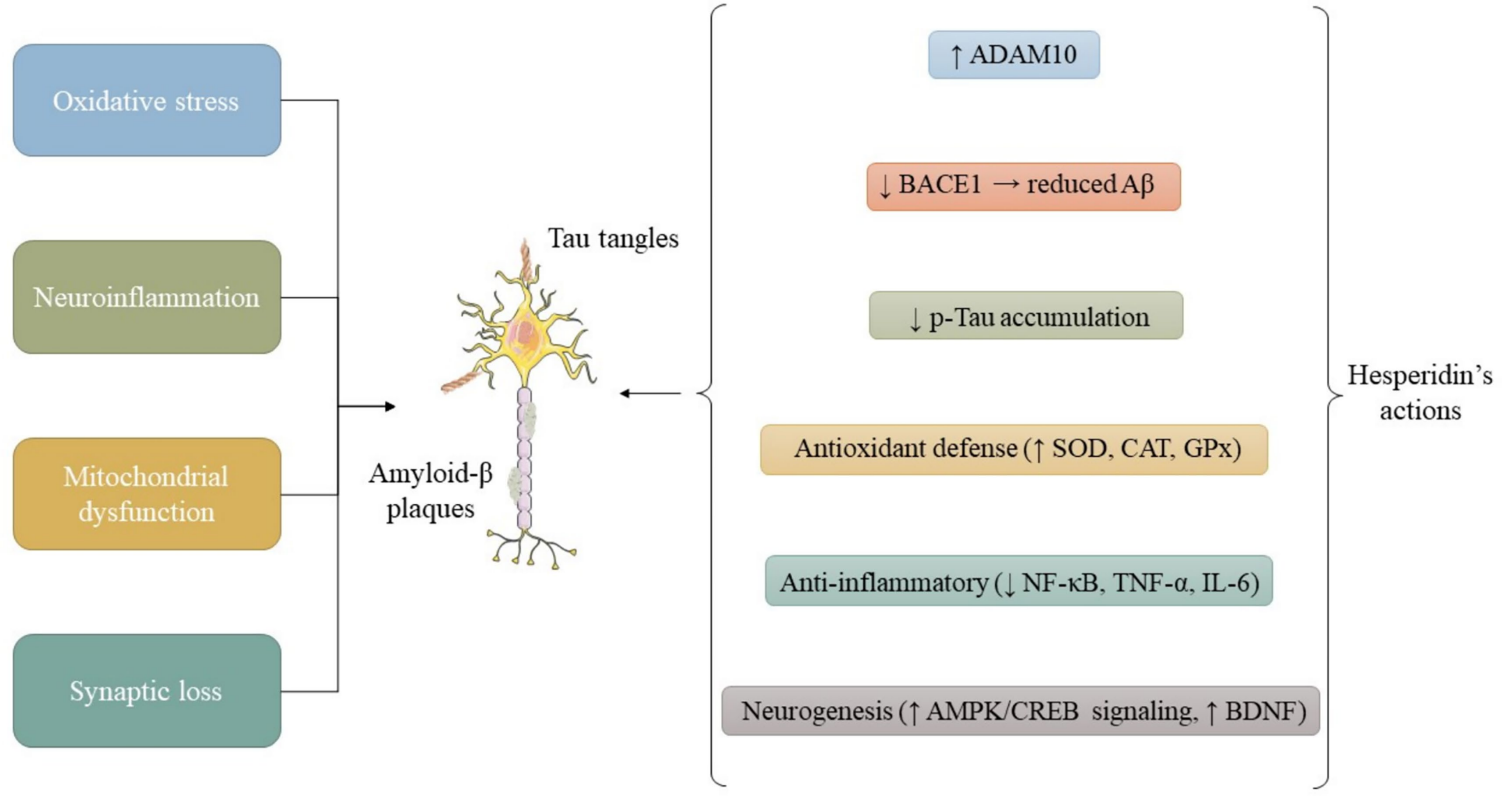
Schematic representation of the primary molecular pathways through which HSP confers neuroprotection. HSP attenuates oxidative stress by scavenging ROS and activating the Nrf2 signaling cascade, leading to up-regulation of antioxidant enzymes such as SOD, CAT, and GPX. In parallel, HSP inhibits NF-κB–dependent cytokine release, preserves mitochondrial membrane potential, and promotes autophagy via modulation of the PI3K/Akt/mTOR axis. Together, these effects sustain cellular homeostasis and synaptic function. Abbreviations: HSP, hesperidin; ROS, reactive oxygen species; Nrf2, nuclear factor erythroid 2–related factor 2; SOD, superoxide dismutase; CAT, catalase; GPX, glutathione peroxidase; NF-κB, nuclear factor kappa-B; PI3K, phosphatidylinositol-3-kinase; Akt, protein kinase B; mTOR, mechanistic target of rapamycin.

Beyond its general antioxidant and anti-inflammatory actions (see Mechanisms of Action), HSP exhibits disease-specific mechanisms, as summarized below (Figure 1).

Preclinical research elucidates its underlying mechanisms of action and assesses its safety profile, while clinical trials systematically examine its therapeutic efficacy in human subjects. Gaining a comprehensive understanding of HSP’s therapeutic potential may facilitate the development of safer and more accessible treatment modalities for AD, thereby addressing an urgent requirement in the management of this debilitating neurodegenerative condition (Table 2). A comprehensive investigation assessed the neuroprotective properties of HSP, a citrus-derived flavonoid, alongside DRB, an inhibitor of casein kinase 2 (CK2), within an *in vitro* model of AD utilizing differentiated SH-SY5Y neuronal cells subjected to amyloid-beta (Aβ1-42) exposure. Both agents demonstrated efficacy in attenuating markers indicative of AD pathology by upregulating ADAM10 and downregulating BACE1 gene expression, thereby diminishing levels of Aβ1-42 and phosphorylated Tau, as well as mitigating neuronal apoptosis. Furthermore, DRB was found to lower CK2α gene expression, implicating CK2 as a prospective therapeutic target. These findings collectively suggest that HSP and DRB effectively inhibit amyloidogenic pathways and confer neuroprotection; however, additional research is warranted to validate these observations (56). AD induces significant memory impairment and cognitive deterioration, which is, in part, attributable to oxidative stress that inflicts damage upon neuronal cells. A separate investigation assessed the antioxidative properties of morin and HSP utilizing a rat model of AD that was induced through administration of streptozotocin. The rats that received treatment with morin and/or HSP exhibited enhancements in memory performance during behavioral assessments and demonstrated a reduction in indicators of oxidative and nitrosative stress in comparison to their untreated counterparts suffering from AD. The synergistic application of both agents augmented the organism’s antioxidant defenses and mitigated detrimental oxidative injury, thereby implying that morin and HSP may confer neuroprotective advantages against memory deficits associated with AD (57).

**Table 2:** The beneficial effects of hesperidin on brain health in Alzheimer’s diseases.

Dosage of hesperidin	Duration of study	Model (*In vitro* or in vivo)	Main effects	Mechanism of effect	Ref
25 μM and 50 μM	24 h pre-treatment (effects observed at 24 and 48 h)	*In vitro* (differentiated SH-SY5Y cells treated with 20 μM Aβ1-42 to mimic AD)	- Increased ADAM10 gene expression (non-amyloidogenic pathway) - Decreased BACE1 gene expression (amyloidogenic pathway) - Reduced Aβ1-42 levels - Reduced p-Tau (T181) levels - Decreased Bax/Bcl-2 ratio (anti-apoptotic effect)	- Inhibition of amyloidogenic pathway and activation of non-amyloidogenic pathway - Inhibition of neuronal apoptosis	(56)
Not explicitly specified (administered for 7 days)	7 days	*In vivo* (STZ-induced AD rat model)	- Reduced oxidative/nitrosative stress markers (NOx, protein carbonyl, AOPP) - Improved antioxidant status (reduced lipid peroxidation) - Ameliorated memory deficits in behavioral tests (MWM and NOR)	Potent antioxidant activity reducing oxidative/nitrosative stress, leading to neuroprotection and cognitive improvement	(57)
Not specified	Not specified	*In vivo* (*Caenorhabditis elegans*, wild-type and AD models CL4176 & CL2006)	- Upregulated acr-16 expression (nicotinic acetylcholine receptor subunit) - Increased autophagy gene expression (lgg-1, bec-1) - Reduced ROS accumulation - Decreased paralysis rate - Extended lifespan and health span	Activation of acr-16 mediated autophagy and antioxidation pathways leading to anti-aging and protection against Aβ toxicity	(58)
Not specified	Not specified	*In vivo* (LPS-induced AD rat model)	- Restored behavioral performance to normal - Enhanced antioxidant defense - Reduced neuronal inflammation - Ameliorated histopathological and ultrastructural brain changes	- Inhibition of HMGB1/TLR4/RAGE pro-inflammatory signaling - Restoration of apoptosis-autophagy balance - Augmentation of antioxidant defenses	(40)
Not specified	Not specified	*In vitro* (neural stem cells from mouse embryos) and *In vivo* (5xFAD AD mouse model)	- Increased proliferation of neural stem cells (NSCs) - Restored hippocampal neurogenesis in 5xFAD mice - Decreased amyloid-beta accumulation - Improved memory function	Activation of AMPK/CREB signaling in NSCs and AMPK/BDNF/TrkB/CREB pathway in hippocampus leading to neurogenesis enhancement and neuroprotection	(59)
Not explicitly stated	48 h	*In vitro* (SK-N-AS cells, AD model induced by Aβ25-35)	Reduced β-amyloid intensity; decreased α-synuclein intensity; neuroprotective effects	Modulation of protein accumulation (β-amyloid, tau, α-synuclein)	(60)
100 mg/kg orally	28 days	*In vivo* (AD-like rat model induced by Scopolamine)	Improved spatial memory; reduced serum TNF-α and IL-1β; increased IL-10; decreased Aβ-42 and AChE activity; reduced oxidative stress (↓ MDA, ↑ GSH); preserved histological structure; decreased p-Tau and GFAP expression; increased synaptophysin expression	Anti-inflammatory, antioxidant; inhibition of AChE activity; reduction of amyloid beta and tau pathology; protection of neuronal structure and synaptic function	(61)
500 nM	Not explicitly stated (*in vitro* assays)	*In vitro* (BACE1 enzyme, amyloid fibril assays)	Complete inhibition of BACE1 enzyme activity; inhibition of amyloid fibril formation; moderate antioxidant activity (ABTS+ assay); strong hydroxyl radical scavenging	High affinity binding to BACE1 active site, blocking substrate access; inhibition of amyloid fibril formation; radical scavenging antioxidant activity	(62)
100 mg/kg (oral)	60 days	*In vivo* (Aluminium chloride-induced cognitive impairment in male Wistar rats)	Prevention of cognitive deficits; reduction of oxidative stress (↓ TBARS, ↑ GSH, ↑ antioxidant enzymes); decreased pro-apoptotic Bax expression; increased anti-apoptotic Bcl-2 expression	Antioxidant activity; anti-apoptotic effects; attenuation of acetylcholinesterase activity and amyloid β biosynthesis markers	(63)
50 or 100 mg/kg/day	16 weeks	*In vivo* (APPswe/PS1dE9 transgenic mice)	Improved learning and memory; improved locomotor activity; increased antioxidant defense; enhanced mitochondrial complex I-IV activities	Inhibition of GSK-3β activity leading to reduced mitochondrial dysfunction; increased antioxidative defense	(42)

AD is characterized by the deleterious accumulation of Aβ, which is intricately associated with autophagy dysfunction and impairment of the α7nAChR. A research demonstrates that HSP, a flavonoid derived from citrus fruits, enhances the expression of acr-16 and autophagy-related genes in *Caenorhabditis elegans*, thereby augmenting autophagic processes and mitigating Aβ toxicity. Furthermore, HSP exhibits significant anti-aging and antioxidant properties, thereby contributing to an increase in lifespan and resilience against stress. The observed benefits are contingent upon the presence of acr-16, thereby underscoring its critical role in mediating the neuroprotective and anti-Aβ effects of HSP (58). AD is characterized by neuroinflammation and the accumulation of β-amyloid plaques, which ultimately result in the degeneration of neuronal cells within the brain. The investigation examined the effects of dapagliflozin and HSP, administered either individually or in combination, on a rat model of AD that was induced by lipopolysaccharide. Both therapeutic interventions demonstrated improvements in behavioral outcomes, enhancement of antioxidant defenses, attenuation of inflammatory responses, and modulation of critical signaling pathways (TLR-4/HMGB1/RAGE) as well as apoptosis within the hippocampal region. The synergistic application of dapagliflozin and HSP exhibited the most pronounced protective effects, leading to the enhancement of cerebral tissue integrity and the mitigation of damage associated with AD. These results imply that this synergistic approach may represent a promising therapeutic strategy for AD by addressing the underlying mechanisms of inflammation, oxidative stress, and autophagy (40).

AD is associated with cognitive decline, in part attributable to diminished neurogenesis. Another investigation elucidated that HSP, a bioflavonoid derived from citrus fruits, enhances the proliferation of neural stem cells via AMPK/CREB signaling pathways, while not influencing their differentiation. In a murine model of AD, HSP reinstated neurogenesis in the hippocampus, mitigated the accumulation of Aβ, and facilitated improvements in memory by activating neuroprotective mechanisms. These findings indicate that HSP represents a promising candidate for both the prevention and therapeutic intervention of AD (59). HSP and naringin, which are flavonoids derived from citrus fruits, were evaluated for their neuroprotective properties in an *in vitro* model of AD utilizing SK-N-AS cells that were subjected to treatment with Aβ25-35. Both flavonoids demonstrated a reduction in the levels of β-amyloid and *α*-synuclein, with naringin exhibiting superior efficacy in diminishing the accumulation of β-amyloid and tau proteins. These findings imply that HSP and naringin possess considerable potential as neuroprotective agents in the context of AD (60).

AD is a chronic age-related neurodegenerative disease characterized by AD is characterized by neurodegeneration, inflammation, and oxidative stress in critical cerebral regions. The present study investigated the neuroprotective properties of HSP within a scopolamine-induced rat model of AD. Rats exhibiting AD manifested cognitive deficits, elevated inflammatory biomarkers, increased amyloid beta levels, heightened acetylcholinesterase activity, oxidative stress, and neuronal tissue impairment. Administration of HSP prior to the onset of symptoms enhanced cognitive function, diminished inflammatory responses and oxidative damage, reduced levels of amyloid beta and tau proteins, and maintained cerebral integrity. These results imply that HSP may possess potential therapeutic efficacy in preventing or alleviating AD-associated neuronal damage (61). AD encompasses numerous detrimental pathways, thereby rendering multi-target therapeutic strategies particularly advantageous. Through the application of an innovative screening methodology, HSP was recognized as a formidable multi-target agent in the context of AD. HSP exhibits a robust inhibitory effect on the activity of the BACE1 enzyme by binding to its active site, thereby obstructing the formation of amyloid aggregates. Additionally, it demonstrates antioxidant properties by effectively scavenging deleterious radicals. These results substantiate the potential of HSP as a natural multi-functional therapeutic candidate for the treatment of AD (62).

The exposure to aluminium has been associated with the progression of AD via mechanisms involving oxidative stress and neuronal apoptosis. The investigation conducted demonstrated that HSP, a bioflavonoid derived from citrus fruits, conferred protection to rats against memory impairment, oxidative injury, and apoptotic processes induced by aluminium chloride, primarily through the enhancement of antioxidant activity and the modulation of pro-apoptotic and anti-apoptotic markers. These results indicate that HSP may represent a promising therapeutic candidate for the treatment of neurodegenerative disorders characterized by oxidative stress and apoptotic phenomena, such as AD (63). Mitochondrial dysfunction and oxidative stress play a significant role in the pathogenesis of AD. A research investigation was conducted to evaluate the effects of HSP in a murine model of AD. Administration of HSP (100 mg/kg) resulted in enhancements in learning, memory, motor activity, antioxidant defense mechanisms, and mitochondrial enzyme functionalities, all while not affecting amyloid-β accumulation. Furthermore, it was observed that HSP treatment led to an increase in GSK-3β phosphorylation, indicating that HSP may facilitate cognitive improvement by ameliorating mitochondrial dysfunction and oxidative injury via the inhibition of GSK-3β (42). In conclusion, both preclinical and clinical studies highlight HSP’s potential as a neuroprotective agent in AD. Its antioxidant and anti-inflammatory effects may help slow disease progression, making it a promising candidate for future therapeutic development. Further research is needed to confirm its efficacy and safety in larger human populations.

### Preclinical and clinical evidence supporting HSP’S neuroprotective effects in PD

**Figure 2:**
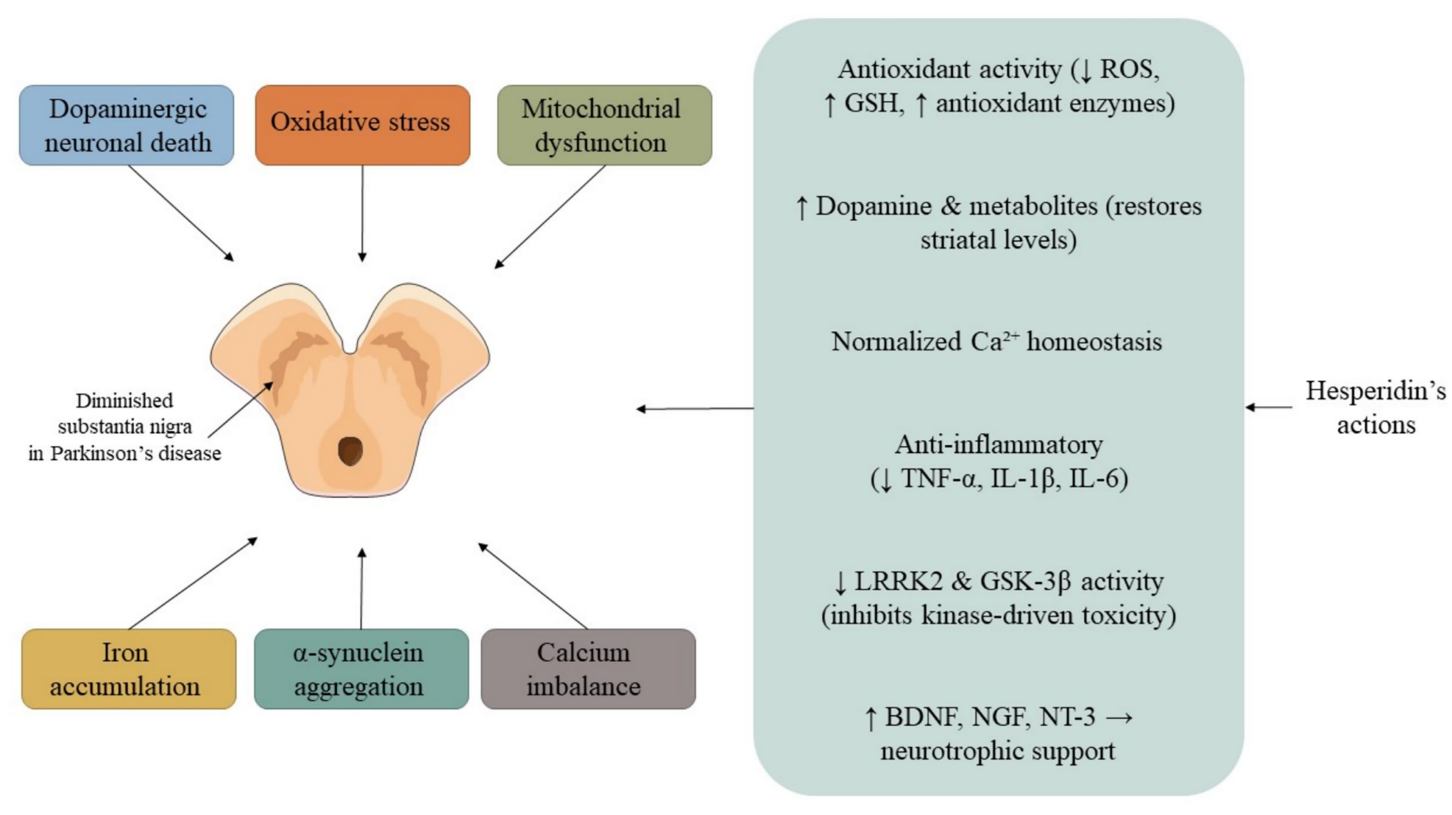
Overview of hesperidin’s major targets in AD and PD. In AD models, HSP modulates APP processing by up-regulating ADAM10 and down-regulating BACE1, reduces Aβ aggregation, and enhances BDNF/CREB signaling to improve synaptic plasticity. In PD, HSP protects dopaminergic neurons through restoration of dopamine levels, inhibition of *α*-synuclein aggregation, and regulation of kinases such as GSK-3β and LRRK2. Both disease contexts share overlapping mechanisms involving oxidative stress reduction, mitochondrial stabilization, and suppression of neuroinflammation. Abbreviations: AD, Alzheimer’s disease; PD, Parkinson’s disease; HSP, hesperidin; APP, amyloid precursor protein; ADAM10, a disintegrin and metalloproteinase domain-containing protein 10; BACE1, β-site APP-cleaving enzyme 1; Aβ, amyloid-β; BDNF, brain-derived neurotrophic factor; CREB, cAMP response element-binding protein; GSK-3β, glycogen synthase kinase-3β; LRRK2, leucine-rich repeat kinase 2.

Beyond its general antioxidant and anti-inflammatory actions (see Mechanisms of Action), HSP exhibits disease-specific mechanisms, as summarized below (Figure 2).

HSP and HSD effectively ameliorated the MPTP-induced alterations in dopaminergic markers and enzyme activities pertinent to Parkinsonism. Collectively, HST and HSP exhibit considerable potential as adjunctive therapies aimed at alleviating the symptoms associated with chemically induced PD (64) (Table 3). *Citrus trifoliata* fruit, which has been historically employed for the management of neurodegenerative conditions, was subjected to an investigation aimed at delineating its bioactive constituents and evaluating its impact on a PD model utilizing rats. The research encompassed five distinct groups, including a control group, a PD group, a PD group treated with L-dopa/Carbidopa, and PD groups administered with two varying dosages of *Citrus trifoliata* extract. The deterioration of brain function was assessed through the application of behavioral assessments, measurement of neurotransmitter concentrations, evaluation of oxidative stress indicators, and histopathological examinations. The extract exhibited motor enhancements that were comparable to conventional PD pharmacotherapy and significantly mitigated oxidative stress by decreasing malondialdehyde and nitric oxide levels while simultaneously reinstating glutathione and SOD concentrations. Furthermore, it led to a reduction in myeloperoxidase activity and reinstated levels of dopamine, GABA, and acetylcholinesterase, which was associated with diminished neuronal apoptosis and inflammation. Metabolite profiling revealed the presence of 40 constituents, predominantly flavonoids such as HSP and naringin. The neuroprotective properties of the extract were ascribed to its antioxidant and anti-inflammatory flavonoids, as evidenced by improvements observed in behavioral, histological, and biochemical parameters (65).

**Table 3:** The beneficial effects of hesperidin on brain health in Parkinson’s diseases, multiple sclerosis, and Huntington’s disease.

Participants	Dosage of hesperidin	Duration of study	Model (*In vitro* or *in vivo*)	Main effects	Mechanism of effect	Ref
Parkinson’s diseases	0.1 and 0.4 mg/g diet	7 days	*In vivo* (*Drosophila melanogaster*)	- Increased lifespan - Improved survival, offspring emergence, climbing ability - Reduced oxidative damage - Normalized antioxidant enzyme activities - Reversed changes in AChE, MAO, TH expression/activity	- Reduced oxidative stress by restoring Keap1/Nrf2 pathway expression - Restored antioxidant enzyme activities (catalase, GST) - Normalized cholinergic and dopaminergic markers (AChE, MAO, TH)	(64)
Parkinson’s diseases	Indirect via *Citrus trifoliata* extract (containing hesperidin) at 50 and 100 mg/kg oral	Not explicitly stated (*in vivo* rat study, likely days to weeks based on behavioral tests)	*In vivo* (Rat model of Parkinson’s disease)	- Motor improvement comparable to L-dopa/carbidopa - Reduced oxidative stress (↓ malondialdehyde, nitric oxide) - Restored antioxidants (glutathione, SOD) - Reduced myeloperoxidase activity - Restored dopamine, GABA, acetylcholinesterase levels - Reduced neuronal apoptosis, microgliosis, and peri-neuronal vacuolation	- Antioxidant and anti-inflammatory effects of flavonoids (hesperidin, naringin) - Replenishment of endogenous antioxidants - Reduction of oxidative stress and neuroinflammation	(65)
Parkinson’s diseases	10 μM	Not explicitly stated (acute exposure, typical Drosophila study likely days)	*In vivo* (*Drosophila melanogaster* Parkinson-like model induced by iron exposure)	- Attenuated motor coordination deficits - Improved memory - Reduced anxiety-like behaviors (grooming, aggressiveness) - Prolonged lifespan - Lowered iron levels in fly heads	- Antioxidant activity - Iron chelation reducing iron accumulation - Protection of dopaminergic system	(66)
Parkinson’s diseases	1, 5, and 10 mM	Not explicitly stated (typical Drosophila study duration)	*In vivo* (*Drosophila melanogaster* expressing human Aβ or α-synuclein)	- Dose-dependent improvement in fecundity, larval motility, climbing ability - No significant change in lifespan	- Neuroprotection against Aβ and α-synuclein toxicity, likely via antioxidant and anti-aggregation effects	(67)
Parkinson’s diseases	Not explicitly stated	Not explicitly stated	*In vitro* (SH-SY5Y cells) and *in vivo* (zebrafish)	- Protection against 6-OHDA-induced neurotoxicity in cells - Improved locomotor behavior in zebrafish	- Downregulation of oxidative stress biomarkers - Downregulation of Parkinson’s-related kinases (LRRK2, GSK-3β) - Reduced apoptosis markers (casp3, casp9) - Modulation of polg gene	(68)
Parkinson’s diseases	Not explicitly stated	Not explicitly stated	*In vitro* (SH-SY5Y cells)	- Protected against 6-OHDA-induced cell damage - Improved cell viability - Reduced intracellular ROS and calcium levels - Restored mitochondrial membrane potential	- Regulation of intracellular calcium homeostasis - Reduction of oxidative stress - Modulation of calcium channel receptor expression	(69)
Parkinson’s diseases	50 mg/kg (oral)	28 days	*In vivo* (C57BL/6 mice with 6-OHDA-induced PD)	- Improved anxiety and depressive-like behaviors - Reduced proinflammatory cytokines (TNF-α, IFN-γ, IL-1β, IL-2, IL-6) - Increased neurotrophic factors (NT-3, BDNF, NGF) - Increased striatal dopamine and metabolite levels - Protection of dopaminergic neurons in substantia nigra	- Modulation of neuroinflammation by reducing cytokines - Enhancement of neurotrophic support - Protection of dopaminergic neurons	(70)
Parkinson’s diseases	10 μM	48 h	*In vivo* (*Drosophila melanogaster*)	- Decreased Fe concentration in fly heads - Restored dopamine levels and AChE activity - Improved motor function - Reduced mortality - Ameliorated oxidative stress and mitochondrial dysfunction	- Iron chelation reducing Fe accumulation - Direct scavenging of reactive species - Activation of antioxidant enzymes (SOD, CAT, GST)	(71)
Parkinson’s diseases	Not applicable (in silico study)	Not applicable (computational)	In silico (molecular docking and dynamics)	Suggested relief of oxidative stress effects during ATP depletion	Binding to SUR1 receptor, potentially protecting neurons from oxidative stress and metabolic imbalance	(72)
Parkinson’s diseases	Not explicitly stated	Not explicitly stated	*In vitro* (human SK-N-SH neuroblastoma cells)	- Reduced apoptosis - Maintained mitochondrial membrane potential - Decreased ROS generation - Prevented GSH depletion - Modulated antioxidant enzyme activities - Regulated apoptosis-related proteins (↓Bax, cyt c, caspases 3 and 9; ↑Bcl-2)	- Antioxidant activity - Maintenance of mitochondrial function - Anti-apoptotic regulation via protein expression	(73)
Multiple sclerosis	Not explicitly stated	21 days	*In vivo* (EAE mouse model, female C57BL/6 mice)	- Inhibited development of experimental autoimmune encephalomyelitis (EAE) - Reduced leukocyte infiltration into CNS - Increased Treg cells and anti-inflammatory cytokines (IL-10, TGF-β) - Decreased Th17 cells and pro-inflammatory cytokines (IL-17, IL-6)	- Modulation of immune response - Upregulation of Treg transcription factor (Foxp3) - Downregulation of Th17 transcription factor (ROR-γt)	(74)
Huntington’s disease	100 mg/kg (oral)	21 days	*In vivo* (rat, QA-induced HD model)	- Attenuated behavioral impairments (locomotor, balance, memory) - Reduced oxidative damage - Improved mitochondrial function - Decreased striatal lesion volume - Modulated TNF-α, caspase-3, BDNF expression	- Antioxidant activity - Microglial modulation (with minocycline)	(75)
Huntington’s disease	50 mg/kg (oral)	14 days	*In vivo* (rat, 3-NP-induced HD model)	- Attenuated behavioral deficits (locomotor activity, grip strength) - Reduced oxidative stress - Restored mitochondrial complex enzyme activities (I, II, IV)	Modulation of nitric oxide (NO) signaling	(76)

Non-motor manifestations such as anxiety and cognitive impairments may serve as early indicators of PD, and interventions aimed at alleviating these symptoms present promising therapeutic alternatives. An investigation centered on HSP assessing its influence on both motor and non-motor manifestations in a *Drosophila melanogaster* model of PD instigated by iron exposure. The flies were categorized into distinct groups, including control, Hsd, L-dopa, iron (Fe), Fe + Hsd, and Fe + L-dopa. Evaluative procedures measured motor coordination, cognitive function, and anxiety-related behaviors such as grooming and aggression. Hsd significantly enhanced motor coordination, cognitive abilities, and anxiety-related behaviors, mitigated monoaminergic deficits, and diminished iron concentrations within the fly heads. Furthermore, Hsd prolonged the lifespan of the flies, surpassing the efficacy of L-dopa treatment. These results imply that Hsd confers protection to the dopaminergic system against iron-induced detriment, inhibits non-motor symptoms, and likely functions through iron chelation in conjunction with its antioxidant characteristics (66). PD and dementia with Lewy bodies exhibit numerous overlapping features, encompassing analogous neurochemical, morphological, and clinical attributes, alongside extensive cortical and limbic *α*-synuclein and amyloid-β pathologies. This investigation assessed the impact of HSP on neurodegeneration precipitated by α-synuclein and amyloid-β in *Drosophila melanogaster*. Employing genetic models that express human Aβ peptide or α-synuclein within neuronal populations, the flies were administered various concentrations of HSP (1, 5, or 10 mM). Neurodegeneration manifested through diminished fecundity, larval motility, climbing proficiency, and overall survival in the affected flies. Nonetheless, the supplementation of HSP enhanced these behavioral parameters in a dose-responsive manner, leading to increased egg production, larval locomotion, and climbing performance when contrasted with untreated specimens. HSP did not exert a statistically significant influence on lifespan. These findings propose HSP as a prospective neuroprotective compound in alleviating neurodegeneration associated with PD and AD (67).

PD is distinguished by the depletion of dopamine within the striatum and the accumulation of Lewy bodies, in conjunction with the upregulation of specific PARK genes and kinases, such as LRRK2 and GSK-3β, which play a role in the formation of tau and alpha-synuclein. HSP was evaluated for its effects against neurotoxicity induced by 6-hydroxydopamine (6-OHDA) in cellular and zebrafish experimental models. HSP conferred protection to SH-SY5Y cells from oxidative stress induced by 6-OHDA and resulted in the downregulation of kinases LRRK2, GSK-3β, and apoptosis-related markers in zebrafish. Furthermore, it enhanced locomotor function that was compromised by 6-OHDA. These findings imply that HSP may serve as a promising therapeutic agent targeting kinases in models of PD (68). PD is characterized by a progressive degeneration of dopaminergic neurons, with existing therapeutic interventions primarily addressing symptomatic relief. Another investigation examined the neuroprotective properties of HSP within a cellular model of PD, emphasizing its influence on calcium (Ca^2+^) homeostasis—a crucial element in the process of neurodegeneration. In the study, SH-SY5Y cells were subjected to 6-OHDA treatment to induce neuronal damage, resulting in elevated intracellular calcium levels, increased ROS concentration, and heightened expression of calcium channel receptors, alongside diminished cell viability and impaired mitochondrial functionality. Administration of HSP effectively mitigated these detrimental impacts by attenuating oxidative stress, reestablishing mitochondrial membrane potential, and normalizing calcium concentration. These results indicate that HSP may confer neuroprotection by modulating calcium homeostasis, thereby underscoring its prospective therapeutic efficacy in the context of PD (69).

An investigation examined the neuroprotective properties of HSP within a murine model of PD, which was induced via the administration of 6-hydroxydopamine (6-OHDA). Following a unilateral striatal lesion, the mice were subjected to oral administration of HSP over a duration of 28 days. Notably, HSP resulted in a significant attenuation of anxiety- and depression-associated behaviors, a reduction in pro-inflammatory cytokine levels (TNF-*α*, IFN-*γ*, IL-1β, IL-2, IL-6), and an augmentation of neurotrophic factors (NT-3, BDNF, NGF) within the striatum. Furthermore, it contributed to an elevation in dopamine levels and its metabolite DOPAC, while also conferring protection to dopaminergic neurons situated in the substantia nigra. These results imply that HSP facilitates neuronal recovery through the modulation of inflammatory processes, enhancement of neurotrophic support, and preservation of dopaminergic functionality in the context of PD (70). Another investigation evaluated the neuroprotective properties of HSP in the context of iron (Fe)-induced neurotoxicity in *Drosophila melanogaster*. Flies subjected to Fe exposure exhibited diminished survival rates, compromised motor function, decreased dopamine concentrations, heightened acetylcholinesterase activity, and increased markers of oxidative stress. Administration of HSP considerably decreased Fe accumulation within the cerebral region, reinstated dopamine and cholinergic functionalities, enhanced motor performance, and diminished mortality rates. Furthermore, it alleviated oxidative stress and mitochondrial dysfunction by neutralizing reactive species and augmenting the activity of antioxidant enzymes. These results suggest that HSP confers neuroprotection against Fe-induced neurodegeneration through various mechanisms pertinent to the pathology of PD (71).

Another investigation delved into the potential mechanisms through which *Valeriana officinalis* extract confers neuroprotective benefits in PD, building upon antecedent discoveries regarding its cytoprotective characteristics. In conjunction with the examination of oxidative stress and mitochondrial dysfunction, a genomic analysis of the substantia nigra was performed utilizing datasets from the Allen Brain Institute. Gene set enrichment analysis and protein-ligand interaction research elucidated key hub genes along with their corresponding proteins, while molecular docking and dynamics simulations indicated that HSP and linarin may mitigate oxidative stress during ATP depletion by interacting with SUR1. Furthermore, valerenic acid and apigenin were also implicated in the attenuation of cortical hyperexcitability by facilitating GABA release from neurons within the substantia nigra (72). An investigation examined the neuroprotective properties of HSP in counteracting rotenone-induced apoptosis within human neuroblastoma SK-N-SH cells, which serve as a pertinent model for PD. Rotenone, a known inhibitor of mitochondrial complex I, incited oxidative stress, a decline in mitochondrial membrane potential, depletion of ATP, an increase in ROS, and apoptotic cell death—characterized by elevated levels of Bax, cytochrome c, and caspases 3 and 9, alongside diminished Bcl-2 expression. Administration of HSP mitigated these detrimental effects by reinstating mitochondrial functionality, attenuating ROS levels, maintaining glutathione concentrations, and normalizing the activity of antioxidant enzymes. Furthermore, it effectively inhibited apoptotic signaling cascades. These findings imply that HSP confers neuroprotection through mechanisms that encompass antioxidant activity, stabilization of mitochondrial integrity, and inhibition of apoptosis (73).

### Preclinical and clinical evidence supporting HSP’S neuroprotective effects in multiple sclerosis (MS)

**Figure 3:**
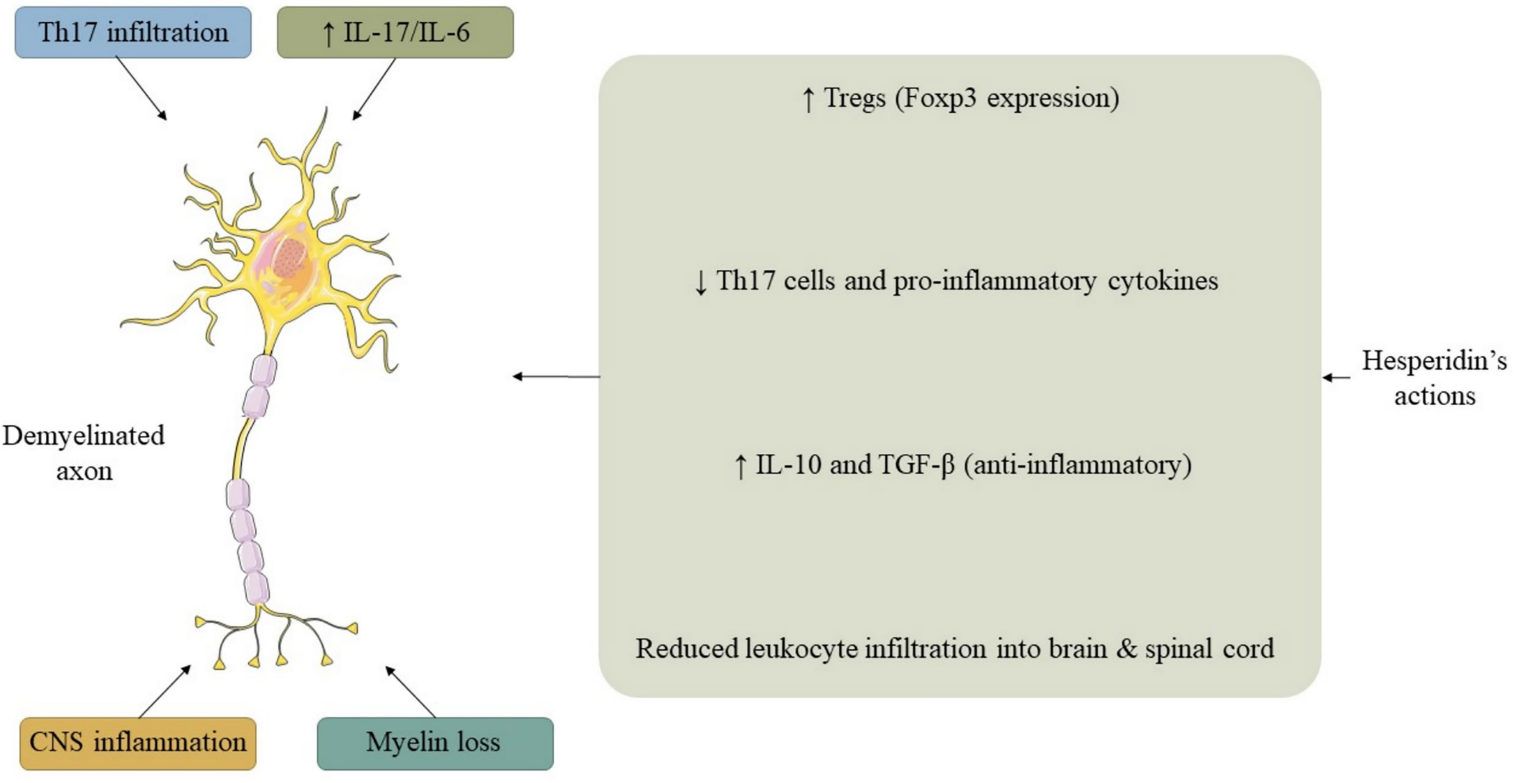
Integrated model summarizing the interconnected effects of HSP on oxidative stress, inflammation, mitochondrial dynamics, and epigenetic regulation. HSP activates the Nrf2/ARE pathway to boost endogenous antioxidant defenses while suppressing NF-κB–mediated inflammatory signaling. Through SIRT1 and AMPK activation, HSP enhances autophagy and mitochondrial biogenesis (via PGC-1α), supporting ATP production and proteostasis. Epigenetic modulation, including histone deacetylation and microRNA regulation, links these processes, coordinating neuronal survival and metabolic balance. Abbreviations: HSP, hesperidin; Nrf2, nuclear factor erythroid 2–related factor 2; ARE, antioxidant response element; NF-κB, nuclear factor kappa-B; SIRT1, sirtuin 1; AMPK, AMP-activated protein kinase; PGC-1α, peroxisome proliferator-activated receptor-*γ* coactivator 1-α; ATP, adenosine triphosphate.

An investigation explored the ramifications of HSP on MS utilizing a murine model of experimental autoimmune encephalomyelitis (EAE). Mice that were immunized with MOG35-55 exhibited characteristic MS-like manifestations, which were markedly alleviated through the administration of HSP. HSP curtailed leukocyte infiltration into both the cerebral and spinal regions, while also altering the immune response by diminishing pro-inflammatory Th17 cells along with their associated cytokines (IL-17, IL-6), concurrently enhancing the presence of regulatory T cells (Tregs) and anti-inflammatory cytokines (IL-10, TGF-β). Analyses of gene expression indicated a downregulation of ROR-γt paired with an upregulation of Foxp3, thereby corroborating this T cell reconfiguration (Table 4). These results imply that HSP attenuates the progression of MS through the modulation of immune responses and the reduction of central nervous system inflammation (74) (Figure 3).

**Table 4:** Comparative summary of preclinical vs. clinical evidence on hesperidin’s neuroprotective effects.

Study type	Model / Population	Key findings	Limitations	Translational relevance	Ref.
Preclinical – In-vivo (AD transgenic mice)	APPswe/PS1dE9 mice, 50–100 mg/kg/day for 16 weeks	Improved learning and memory; enhanced activities of mitochondrial complexes I–IV; reduced oxidative damage; some improvement in locomotor behavior.	Plaque load not significantly changed at lower dose; high dose needed; differences in mouse vs. human metabolism and brain exposure.	Supports potential for mitochondrial and cognitive benefit; yet unknown whether similar doses are safe/effective in humans.	(42)
Preclinical – neurogenesis / NSC + 5xFAD mice	NSCs isolated from mouse embryos + 5xFAD mice model	Hesperidin increased NSC proliferation (via AMPK/CREB), restored neurogenesis, reduced amyloid-β, improved cognition.	Preclinical; high dose; short-term study; effect on differentiation less clear.	Suggests disease-modifying potential, but human studies lacking.	(59)
Clinical – bioavailability/human kinetics	Healthy volunteers, orange juice or modified hesperidin preparations (micronized, enzymatic, etc.)	Treatments such as enzymatic conversion (to hesperetin-7-glucoside), micronization, particle size reduction significantly increase plasma/urine levels, reduce T_max_.	Measured peripheral biomarkers/metabolites, not CNS levels; cognitive or neurological endpoints not studied.	Demonstrates practical methods to improve exposure; need trials measuring brain effects/cognition.	(81)
Clinical – (limited) human trials with cognitive or neurological endpoints	*No large RCTs identified* with neurological disease populations; existing studies mostly in healthy subjects or metabolic biomarkers.	Some improvement in endothelial function, oxidative biomarkers; possibly peripheral cognitive performance in small trials.	Small sample size; short duration; often non-neurological primary endpoints; formulation/dose varies.	Gap remains: human disease trials with neurodegenerative endpoints are needed.	-

### Preclinical and clinical evidence supporting HSP’S neuroprotective effects in Huntington’s disease

This investigation examined the neuroprotective capabilities of HSP, both in isolation and in conjunction with minocycline (a known microglial inhibitor), within a rat model of Huntington’s disease (HD) induced by quinolinic acid (QA). The administration of QA resulted in pronounced behavioral deficits, oxidative stress, mitochondrial dysfunction, an increase in striatal lesion volume, elevated levels of TNF-*α* and caspase-3, and a decrease in BDNF expression. Prolonged treatment with HSP (100 mg/kg) or minocycline (25 mg/kg) administered separately alleviated these detrimental effects, whereas a reduced dosage of HSP (50 mg/kg) alone proved ineffective. Nonetheless, the combination of lower-dose HSP with minocycline markedly augmented neuroprotection. These results imply that the neuroprotective mechanism of HSP encompasses both its antioxidant properties and the modulation of microglial activity (75). Another investigation examined the neuroprotective properties of HSP and naringin in counteracting neurotoxicity induced by 3-nitropropionic acid (3-NP) in a rat model simulating Huntington’s disease (HD). The administration of 3-NP resulted in manifestations analogous to HD, such as compromised motor abilities, weight reduction, oxidative stress, and mitochondrial dysfunction. The prior administration of HSP or naringin led to marked behavioral improvements, a decrease in oxidative injury, and the reinstatement of mitochondrial enzymatic activity. The simultaneous administration of L-arginine (a precursor of nitric oxide) mitigated their neuroprotective efficacy, whereas L-NAME (an inhibitor of nitric oxide synthase) amplified their beneficial effects. These results imply that HSP and naringin confer protection against neurotoxic effects resembling HD, likely via the modulation of the nitric oxide signaling pathway (76).

### Comparative perspective with other flavonoids

While several citrus-derived flavonoids show neuroprotective potential, HSP possesses a distinctive biochemical profile. Its glycosidic form limits passive diffusion, yet microbial hydrolysis to hesperetin enhances CNS penetration. Compared with quercetin and naringenin, HSP/hesperetin exhibit unique epigenetic activity, influencing DNA methylation and histone acetylation, as well as strong modulation of autophagy pathways. These features may partly explain the consistent improvements in synaptic and mitochondrial parameters observed across preclinical models. Future studies directly comparing these compounds could clarify structure–activity relationships and inform formulation strategies (77–80).

## Limitations

Despite robust preclinical data, translation to humans remains uncertain. Differences in pharmacokinetics, metabolism, and blood–brain barrier permeability substantially affect HSP’s systemic and CNS exposure. Moreover, most experimental models simplify disease mechanisms and use supraphysiologic doses that may not be achievable in humans. These translational barriers should temper interpretation of animal results and highlight the need for rigorous dose-finding and formulation studies before clinical extrapolation.

Despite encouraging preclinical data, the current evidence base remains preliminary. The majority of studies are small, non-replicated, and heterogeneous in design, making it difficult to directly compare outcomes or estimate true effect sizes. The majority of extant investigations have been executed *in vitro* or through animal models, which may not adequately represent the intricate pathophysiology associated with human neurodegenerative disorders. Another important limitation concerns interspecies variability in pharmacokinetics and metabolism. Rodents and other preclinical models often exhibit faster absorption and higher relative bioavailability of HSP and its metabolites than humans, which can exaggerate apparent efficacy. Furthermore, many animal studies use supra-physiological doses and simplified models of neurodegeneration that do not fully capture the chronic, multifactorial nature of human disease. These factors collectively complicate direct extrapolation of preclinical outcomes to clinical scenarios and emphasize the need for careful dose translation and pharmacokinetic modeling in future studies.

The bioavailability of HSP within the human body poses a considerable challenge owing to its suboptimal absorption and rapid metabolic degradation, which may ultimately restrict its therapeutic effectiveness. Moreover, there exists an absence of standardized dosing regimens and formulations across various studies, complicating the process of result comparison and the establishment of optimal treatment protocols. The long-term safety profile and potential adverse effects of HSP supplementation have not been rigorously assessed in extensive human clinical trials. Furthermore, the inherent heterogeneity of neurodegenerative disorders—characterized by varying etiologies and rates of progression—complicates the evaluation of HSP’s efficacy among distinct patient cohorts. Lastly, the precise molecular targets and epigenetic mechanisms associated with HSP necessitate further elucidation to enhance the comprehension of its neuroprotective pathways. Addressing these constraints through rigorous clinical investigations and the standardization of methodologies is imperative to fully realize HSP’s therapeutic potential.

## Conclusion and key messages

By modulating epigenetic markers such as DNA methylation and histone acetylation, HSP regulates genes involved in neuronal survival and plasticity. By influencing these epigenetic pathways, HSP fosters a conducive molecular environment that underpins cerebral health throughout the aging process. In addition to its role in epigenetic regulation, HSP plays a significant role in sustaining cellular homeostasis by mitigating oxidative stress and inflammation—two primary contributors to neurodegenerative pathology. The restoration of cellular homeostasis serves to protect neurons from potential damage and dysfunction. In addition, research has demonstrated that HSP can enhance synaptic function and plasticity, both of which are crucial for processes such as learning, memory retention, and overall cognitive resilience. The integration of these effects contributes significantly to its robust neuroprotective profile, as evidenced by numerous preclinical models examining neurodegeneration. Notwithstanding this encouraging evidence, the corpus of clinical studies involving human subjects remains scant, underscoring the necessity for rigorously designed trials to substantiate the therapeutic advantages of HSP and to ascertain optimal dosing regimens and delivery mechanisms. Overall, HSP represents a promising natural compound with multifaceted actions that may help address neurodegenerative disorders. Central insights derived from this review accentuate HSP’s ability to modulate gene expression via epigenetic pathways, bolster cellular health by alleviating oxidative and inflammatory stressors, and uphold synaptic integrity to sustain cognitive function. This extensive neuroprotective profile underscores the critical necessity for translational research aimed at fully leveraging the therapeutic potential of HSP within clinical frameworks, as well as the development of innovative interventions capable of delaying or preventing the advancement of debilitating neurodegenerative disorders.

## Future directions

Future investigations concerning HSP should emphasize rigorously structured clinical trials to assess its safety, bioavailability, and efficacy in human populations afflicted by age-related and neurodegenerative disorders. In light of HSP’s diverse mechanisms of action, inquiries into optimal dosing regimens, delivery methodologies (inclusive of nanoformulations or combined therapies), and long-term implications are imperative to translate preclinical achievements into clinical applications. Furthermore, a thorough exploration of the specific epigenetic modifications induced by HSP will augment our comprehension of its regulatory effects on gene networks pertinent to neuroprotection and cognitive resilience. The integration of multi-omics methodologies, including epigenomics, transcriptomics, and proteomics, has the potential to reveal novel biomarkers for monitoring HSP’s therapeutic impacts and patient responsiveness. Research endeavors should further investigate the interactions between HSP and various neurotherapeutic agents, as well as consider lifestyle determinants such as dietary habits and physical activity, in order to formulate synergistic therapeutic strategies. Ultimately, broadening the scope of research to encompass a variety of neurodegenerative models as well as age-associated cognitive impairments will elucidate the extent of HSP’s neuroprotective capabilities and promote the development of personalized medical interventions. In summary, enhancing our comprehension of the mechanisms through which HSP operates and its clinical applicability will be crucial in positioning it as a plausible natural intervention for the prevention or attenuation of neurodegenerative processes.
